# Supplementary material for: Evaluation of water conservation function in the Xiongan New Area based on the comprehensive index method
Source: PLoS One. 2020 Sep 10;15(9):e0238768. doi: 10.1371/journal.pone.0238768 (PMC7482998; doi:10.1371/journal.pone.0238768)
Supplement: Attachment — Submitted filename: Response to reviewers.docx [file pone.0238768.s002.docx]

**Our Responses to the comments on the Manuscript**

**Dear Editors and Reviewers:**

Thank you for your letter and for the reviewers’ comments concerning our manuscript entitled “Evaluation of Water Conservation Functions in the Xiongan New Area Based on a Comprehensive Index Method” (No.PONE-D-19-19149).Those comments are all valuable and very helpful for revising and improving our paper, as well as the important guiding significance to our researches. We have studied comments carefully and have made correction which we hope meet with approval. Revised portion are marked in red in the paper. The main corrections and responds to the reviewers’ comments are as follows.

**Editor**

1. Please ensure that your manuscript meets PLOS ONE's style requirements, including those for file naming.

**Response:** We are sure that our manuscript meets PLOS ONE's style requirements.

2. Thank you for stating the following financial disclosure:

"The funders had no role in study design, data collection and analysis, decision to publish, or preparation of the manuscript".

**Response:** We agree with you. We have stated that "The funders had no role in study design, data collection and analysis, decision to publish, or preparation of the manuscript".

The information of funding institutions, relevant grant numbers and authors who were grant recipients are as follows:

(1) National Key Research and Development Program of China, 2017YFB0504102, Adu Gong

(2) National Key Research and Development Program of China, 2017YFC1502402, Adu Gong

(3) National Natural Science Foundation of China, 41671412, Adu Gong

3. Please ensure that you refer to Figures 1, 3 and 4 in your text as, if accepted, production will need this reference to link the reader to the figure.

**Response:** We have made correction according to the Editor’s suggestion. We have checked and revised Figures 1, 3 and 4 in our text so that readers can link to the figures.

4. We note you have included tables to which you do not refer in the text of your manuscript. Please ensure that you refer to in your text; if accepted, production will need this reference to link the reader to each Table.

**Response:** We have made correction according to the Editor’s suggestion. We have checked and revised Tables 1, 2 and 8 in our text so that readers can link to the Tables.

**Reviewer #1:**

1. Introduction. There has already been a large amount of literatures discussing this topic, there is a need to better elaborate the contribution of the work to the existing literature. Information of the models existing and the differences among these and the novel model, what are the advantages and disadvantages of the approach, usage, develop and in general why your model is better, lacks of arguments in the introduction section. Please, be more critical in addressing the research gap. What is the contribution of the paper to the literature? Emphasize these aspects already in the literature reviews to make paper attractive for readers. Some references should be included. e.g., (i) Evaluating sustainability of water-energy-food (WEF) nexus using an improved matter-element extension model: A case study of China. Journal of Cleaner Production. 2018;202:1097-106 (ii) Decoupling analysis of economic growth from water use in City: A case study of Beijing, Shanghai, and Guangzhou of China. Sustainable cities and society. 2018;41:86-94. (iii) Evaluating water resource sustainability in Beijing, China: Combining PSR model and matter-element extension method. Journal of Cleaner Production. 2019;206:171-9.

**Response:** We have rewritten this part according to the Reviewer’s comments. We have rewrote the Introduction section by reading a large amount of literature. We have added the significance of studying the water conservation function, the existing research methods, the advantages and disadvantages of each method, the reasons for choosing this research method, and also explained the significance of Xiongan New Area was selected as the study area. In addition, we have added many classic articles on water resources according to Reviewer’s advice.

2. Study area and data sets. Few articles have made this part a separate section. Please read the latest published papers in PLOS ONE carefully and re-arrange the section according to the format of the previous paper in PLOS ONE. It is recommended to merge the Study areas and data sets with the Methodology as one section.

**Response:** We have made correction according to the Reviewer’s suggestion. I have read the latest published papers in PLOS ONE carefully and merged the Study areas and data sets with the Methodology as one section.

3. Methodology. It is recommended to merge the Study areas and data sets with the Methodology as one section.

**Response:** We have made correction according to the Reviewer’s suggestion. We merged the Study areas and data sets with the Methodology as one section.

4. Results and analysis. It is seemed that there is only a results statement, no discussion or analysis. I should suggest to add an discuss what your findings are different from the past works.

**Response:** We have made correction according to the Reviewer’s suggestion. Firstly, we revised the results and analysis sections to make the results clearer and the presentations smoother. In addition, we have added the discussion section, mainly to analyze the reasons for the results, and pointed out the advantages and limitations of the research method compared with other methods. Please refer to the discussion section for details.

5. Conclusion. This section is well written. I would suggest to write more explicitly the main findings of this study.

**Response:** We have made correction according to the Reviewer’s suggestion. We have revised the conclusions to highlight the findings of this paper.

6. References. Please check the references in the text and the list; References should be revised and upgraded.

**Response:** We have made correction according to the Reviewer’s suggestion. We have checked every reference carefully, and since some new literatures were added, the references were updated.

7. Grammar check. There are still some occasional grammar errors through the manuscript especially the article ''the'', ''a'' and ''an'' is missing in many places, please make a spellchecking in addition to these minor issues.

**Response:** We have made correction according to the Reviewer’s suggestion. I have revised the entire manuscript word for word, and I have made a spellchecking. I did find some grammatical errors and made corrections.

**Reviewer #2:**

The article has no innovations in terms of ideas and data processing, the data is not new enough, the pictures in the text are not clear enough, the references are not new, and the guiding significance is not strong.

**Response:** We have made correction according to the Reviewer’s suggestion. (1)We agree with the opinion of reviewer. This study cannot be regarded as a method innovation in a strict sense. The method of this paper was improved on the“Technical Criterion for Ecosystem Status Evaluation”issued by Ministry of Environmental Protection of the People’s Republic of China, and added some new indicators, considering the actual situation of the study area and the principle of water conservation. In addition, Xiongan New Area is deemed another new national zone of significance after the Shenzhen Special Economic Zone and the Shanghai Pudong New Area. It is a millennium plan and a national event. Baiyangdian Wetland is the largest lake wetland in the North China Plain, which is known as the “kidney of North China”，and most of which are located in Xiongan New Area. Studying the trend of water conservation function in Xiongan New Area during the past ten years is conducive to supporting the environmental assessment of government and better protecting Baiyangdian wetland. (2) Since the land use/cover data is only updated to 2015, in order to ensure the uniformity of the data, this study does not include other auxiliary data, and new data will be added in the future research.

(3)We have corrected all the pictures in the article to achieve the optimum clarity of the pictures.

(4) We have revised and updated the references and added plenty of the latest literature.
