# Supplementary material for: Evaluation of water conservation function in the Xiongan New Area based on the comprehensive index method
Source: PLoS One. 2020 Sep 10;15(9):e0238768. doi: 10.1371/journal.pone.0238768 (PMC7482998; doi:10.1371/journal.pone.0238768)
Supplement: Attachment — Submitted filename: Response to reviewers.docx [file pone.0238768.s003.docx]

**Our Responses to the comments on the Manuscript**

**Dear Editors and Reviewers:**

Thank you for your letter and for the reviewers’ comments concerning our manuscript entitled “Evaluation of Water Conservation Functions in the Xiongan New Area Based on a Comprehensive Index Method” (No.PONE-D-19-19149).Those comments are all valuable and very helpful for revising and improving our paper, as well as the important guiding significance to our researches. We have studied comments carefully and have made correction which we hope meet with approval. Revised portion are marked in red in the paper. The main corrections and responds to the reviewers’ comments are as follows.

**Reviewer #1:**

The authors have incorporated comments from the first round of review. My concerns from my previous review have been addressed. I would recommend the paper to be accepted for publication.

**Response:** Thanks to the recognition of the experts, we carefully read and revised our manuscript, hoping to reach the level of publication.

**Reviewer #2:**

1. What's the innovation of this paper? the significance? It's still not clear. The authors should summarized and showed it in a certain part in the paper, maybe in the end of the introduction.

**Response:** Thanks to the reviewer's advice. We have summarized the innovation of this paper in the end of the introduction according to the Reviewer’s suggestion.

2. The language should be more carefully corrected throughout the whole manuscript.

Response: Thanks to the reviewer's advice. We have corrected the language throughout the whole manuscript according to the Reviewer’s suggestion. In addition, the language of our manuscript have been refined and polished by a professional editing company.

3. Line 31-44, this paragraph may be shortened to just show the significance of this study.

**Response:** Thanks to the reviewer's advice. We have shorted this paragraph according to the Reviewer’s suggestion and highlighted the importance and significance of water conservation function evaluation.

4. Line 45-63, the use of RS, the authors take so much sentences on the use of RS, Does it the main method or the features of this paper? What's more, so many literatures were just listed here. And it is the same with next paragraph. Another paragraph should be added to show how this paper to tackle this problems.

**Response:** Thanks to the reviewer's advice. We have made correction according to the Reviewer’s suggestion. (1) We originally intended to highlight the main research methods of ecological function evaluation, but now we feel that they do not have much relationship with the article, so we deleted line 45-63 directly according to the Reviewer’s opinion. (2) The next paragraph mainly describes the assessment methods of water conservation function and introduces a widely used model, InVEST. However, this method is not the method adopted in this paper, so the description of this paragraph is simplified according to the opinions of expert. (3) In the next paragraph, we added the application and advantages of the comprehensive index method used in this paper.

5. Line 120-121, Why data in April and May were chosen? and What about data in summer or the other season? and Why it wasn't in the same month?

**Response:** Thanks to the reviewer's advice. Due to the poor data quality of Landsat in May of 2005 in Xiongan New Area, the data of April which is the latest with the dates of 2010 and 2015 were originally selected. Although there is a certain deviation in time, the impact is not very big. Landsat data were used to calculate the TVDI index, which is jointly determined by NDVI and LST. The result shows that the drought was the severest in 2010, the least severe in 2015 and moderate in 2005. In addition, from the meteorological data analysis, the degree of drought difference in April and May is not big. The data of May are selected as the representative mainly because the Xiongan New Area is located in the north China plain, with a large amount of cultivated land, and winter wheat are mainly crops. May is the best period for the growth of winter wheat, which is conducive to the calculation of various indexes.

6. Line 130-131, What's the relation between the data of all the time nodes?

**Response:** Thanks to the reviewer's advice. The land cover data used in this study is the dataset of China's land use types released by the Chinese Academy of Sciences. The data were classified based on Landsat images. The late 1970s and late 1980s were built in intervals of 10 years, and were subsequently released every five years to analyze the change rules of China's land use.

7. Line 159, What's GI stand for?

**Response:** Thanks to the reviewer's advice. We have consulted a large number of literatures. Some people called this method "rank correction analysis method" and others "order relation analysis method". We referred to the opinions of most people and finally adopted "rank correction analysis method". "GI" is short for this method, but it is not authoritative, so we delete it this time.

8. Line 193, What's the difference between woodland and forestland? they both appeared in this paper.

**Response:** Thanks to the reviewer's advice. Due to our negligence, woodland and forestland both appeared in the article at the same time. We have carefully revised and changed all woodland into forestland.

9. Too many conclusions were made. They should be further summarized to show the main conclusion directly connected to the main aim of this paper.

**Response:** Thanks to the reviewer's advice. We have summarized the conclusion to highlight the aim of this paper according to the Reviewer’s suggestion.
