# Supplementary material for: Evaluation of water conservation function in the Xiongan New Area based on the comprehensive index method
Source: PLoS One. 2020 Sep 10;15(9):e0238768. doi: 10.1371/journal.pone.0238768 (PMC7482998; doi:10.1371/journal.pone.0238768)
Supplement: Attachment — Submitted filename: Response to reviewers.docx [file pone.0238768.s004.docx]

**Our Responses to the comments on the Manuscript**

**Dear Editors and Reviewers:**

Thank you for your letter and for the reviewers’ comments concerning our manuscript entitled “Evaluation of Water Conservation Functions in the Xiongan New Area Based on a Comprehensive Index Method” (No.PONE-D-19-19149).Those comments are all valuable and very helpful for revising and improving our paper, as well as the important guiding significance to our researches. We have studied comments carefully and have made correction, which we hope meet with approval. Revised portion are marked in red in the paper. The main corrections and responds to the reviewers’ comments are as follows.

**Reviewer #1:**

1. “Compared with the InVEST model, the comprehensive index method is more convenient, and the parameters and weights can be adjusted according to the characteristics of the research area, which improves evaluation accuracy.” How can this be proved？please explain more.

**Response:** Thanks to the reviewer's advice. There are many methods for evaluating water conservation functions, and each method has its advantages and limitations. We can't explain which method is better. We think that the expression of this paragraph is not very appropriate. We can only explain that the comprehensive index method used in this article is more convenient than the InVEST model. The comprehensive index method does not need localized calibration as the InVEST model. In addition, different index factors can be selected according to the characteristics of the study area. Thus, we revised the paragraph to “Each method has its advantages and limitations. In this paper, we choose the comprehensive index method to evaluate water conservation functions. This method more convenient than the InVEST model, which does not need localized calibration of model parameters, and the parameters can be adjusted according to the characteristics of the research area whenever necessary”

In addition, we have referred to a lot of literatures to supple the advantages and limitations of the InVEST model. Supplementary instructions are as follows:

The InVEST (Integrated Valuation of Ecosystem Services and Tradeoffs) model was jointly developed by Stanford University, the Nature Conservancy and the World Wide Fund for Nature to assess ecosystem service functions. InVEST model has multiple modules, covering almost all ecological service functions. The water conservation module is an important part of the InVEST model, which is based on the water balance method. InVEST model has been successfully applied to Willamette River in the USA^[1]^, Cote d'Ivoire in West Africa^[2]^, Francoli basin in northeastern Spain^[3]^. Chinese scholars successfully applied the InVEST model to Beijing ^[4]^, Dujiangyan^[5]^, Sanjiangyuan District^[6,7]^, Qinling Mountains^[8]^, Fujian^[9]^, Dongting Lake^[10]^ and other regions in China. The applicability of InVEST model and the local suitability of parameters are the key to the reliability of the model. However, the calculation methods and parameters in the InVEST model are mostly based on American standards. When applied to the regions of China, it is necessary to establish a constantly improving database that conforms to the features of regional ecosystem service functions, and use the measured data to correct the model parameters. In addition, data acquisition is not easy and is sensitive to data changes. Furthermore, the InVEST model takes the land use type as a unit, and does not consider the topography and other factors.

[1] Erik Nelson, Guillermo Mendoza, James Regetz, Stephen Polasky, Heather Tallis, DRichard Cameron, et al. Modeling multiple ecosystem services, biodiversity conservation, commodity production, and tradeoffs at landscape scales. Frontiers in Ecology and the Environment.2009; 7(1):4-11.

[2] Leh, M.D.K., Matlock, M.D., Cummings, E.C., Nalley, L.L. Quantifying and mapping multiple ecosystem services change in West Africa. Agriculture, Ecosystems and Environment. 2013; 165, 6-18.

[3] Marquès M, Bangash RF, Kumar V, Sharp R, Schuhmacher M. The impact of climate change on water provision under a low flow regime: a case study of the ecosystems services in the Francoli river Basin. Journal of Hazardous Materials.2013; 263:224-32.

[4] Yu XX, Zhou B, Lv XZ, Yang ZG. Evaluation of Water Conservation Function in Mountain Forest Areas of Beijing Based on InVEST Model. Scientia Silvae Sinicae. 2012; 48(10):1-5.

[5] Fu B, Xu P, Wang YK, Peng Y, Ren J. Spatial Pattern of Water Retetnion in Dujiangyan County. Acta Ecologica Sinica. 2013; 33(3):0789-97.

[6] Pan T, Wu SH, Dai EF, Liu YJ. Spatiotemporal variation of water source supply service in Three Rivers Source Area of China based on InVEST model. Chinese Journal of Applied Ecology.2013;24(1):183-9.

[7] Lv LT, Ren TT, Sun CZ, Zheng DF, Wang H. Spatial and temporal changes of water supply and water conservation function in Sanjiangyuan National Park from 1980 to 2016. Acta Ecologica Sinica. 2020; 40(3):993-1003.

[8] Fan YN, Liu K, Chen SS, Yuan J G. Spatial pattern analysis on water conservation functionality of land ecosystem in Northern slope of Qinling Mountains. Bulletin of Soil and Water Conservation. 2017; 37(2): 50-6.

[9] Wang BS, Chen HX, Dong Z, Zhu W, Qiu QY, Tang LN. Impact of land use change on the water conservation service of ecosystems in the urban agglomeration of the Golden Triangle of Southern Fujian, China, in 2030. Acta Ecologica Sinica. 2020;40(2) :484-98.

[10] Hu WM, Li G, Gao ZH, Jia GY, Wang ZC, Li Y. Assessment of the impact of the Poplar Ecological Retreat Project on water conservation in the Dongting Lake wetland region using the InVEST model. Science of The Total Environment. 2020.

2. "2015" is missing in the third figure, which should be the same as the first two figures, such as Fig.3 and fig.8

**Response:** Thanks to the reviewer's advice. Due to our negligence, "2015" was missing in some figures. We have checked and revised all figures carefully.
